# Sequential Infiltration Synthesis into Maltoheptaose and Poly(styrene): Implications for Sub-10 nm Pattern Transfer

**DOI:** 10.3390/polym14040654

**Published:** 2022-02-10

**Authors:** Anette Löfstrand, Alexei Vorobiev, Muhammad Mumtaz, Redouane Borsali, Ivan Maximov

**Affiliations:** 1 NanoLund and Solid State Physics, Lund University, SE-221 00 Lund, Sweden; 2 Division for Materials Physics, Department of Physics and Astronomy, Uppsala University, SE-751 20 Uppsala, Sweden; alexey.vorobiev@physics.uu.se; 3 Université Grenoble Alpes, CNRS, CERMAV, 38000 Grenoble, France; muhammad.mumtaz@cermav.cnrs.fr (M.M.); redouane.borsali@cermav.cnrs.fr (R.B.)

**Keywords:** block copolymer, sequential infiltration synthesis, hybrid material, neutron reflectometry

## Abstract

Vapor phase infiltration into a self-assembled block copolymer (BCP) to create a hybrid material in one of the constituent blocks can enhance the etch selectivity for pattern transfer. Multiple pulse infiltration into carbohydrate-based high-*χ* BCP has previously been shown to enable sub-10 nm feature pattern transfer. By optimizing the amount of infiltrated material, the etch selectivity should be further improved. Here, an investigation of semi-static sequential infiltration synthesis of trimethyl aluminum (TMA) and water into maltoheptaose (MH) films, and into hydroxyl-terminated poly(styrene) (PS-OH) films, was performed, by varying the process parameters temperature, precursor pulse duration, and precursor exposure length. It was found that, by decreasing the exposure time from 100 to 20 s, the volumetric percentage on included pure Al_2_O_3_ in MH could be increased from 2 to 40 vol% at the expense of a decreased infiltration depth. Furthermore, the degree of infiltration was minimally affected by temperature between 64 and 100 °C. Shorter precursor pulse durations of 10 ms TMA and 5 ms water, as well as longer precursor pulses of 75 ms TMA and 45 ms water, were both shown to promote a higher degree, 40 vol%, of infiltrated alumina in MH. As proof of concept, 12 nm pitch pattern transfer into silicon was demonstrated using the method and can be concluded to be one of few studies showing pattern transfer at such small pitch. These results are expected to be of use for further understanding of the mechanisms involved in sequential infiltration synthesis of TMA/water into MH, and for further optimization of carbohydrate-based etch masks for sub-10 nm pattern transfer. Enabling techniques for high aspect ratio pattern transfer at the single nanometer scale could be of high interest, e.g., in the high-end transistor industry.

## 1. Introduction

The development of high technology devices often demands improved resolution in lithography and pattern transfer techniques. By infiltrating metal–organic molecules into soft material, so-called hybrid materials are created [1] which can serve as etch masks with improved etch selectivity during pattern transfer [2]. Block copolymer (BCP) lithography is a highly promising technique to enable sub-10 nm pattern transfer—it has a potential to generate highly dense patterns over large areas at a low cost [3]. Such small sizes could be of interest in the high technology industry, e.g., for high-end transistor fabrication, where a high density of low-cost transistors is especially attractive [4]. Utilizing high immiscibility of polymers, so-called high-*χ* materials [3], patterns of sub-10 nm pitch can be created from diblock copolymers by exposing it to solvent vapor [5,6]. By careful choice of parameters, the BCP may, e.g., self-assemble into lamellae, or into cylinders or spheres of one block in a hexagonal lattice, surrounded by a matrix of the other block [2].

Hybrid materials can be created when vapors of organo-metallic molecules are infiltrated into a polymer, a process often referred to as vapor phase infiltration (VPI) or sequential infiltration synthesis (SIS) [7]. There are several techniques, including static, semi-static, and dynamic infiltration. In static infiltration, the chamber inlet and outlet are both temporarily closed to contain the introduced molecules. In semi-static infiltration, the outlet is temporarily closed, but the inlet kept open, whereas in dynamic infiltration both inlet and outlet are constantly left open. The molecules may then adsorb and react with functional groups within the polymer [1]. Introduction of subsequent vapors of oxygen containing molecules, such as water, may then adsorb and react, to eventually form a metal oxide. This process has previously been studied for a number of materials, such as of the precursor trimethyl aluminum (TMA) into poly(metyl methacrylate) (PMMA) [8], poly(2-vinylpyridine) (P2VP) [9], hydroxyl-terminated poly(styrene) (PS-OH) [10], and maltoheptaose (MH) [10]. Functional groups that have been identified to react with TMA precursor are, e.g., hydroxyl groups (-OH) [11], carbonyl groups (>C=O) [8], and pyridines [12]. Some of the characterization techniques for SIS are Fourier transform infrared spectroscopy (FTIR) [13], quartz crystal microbalance (QCM) [14], scanning transmission electron microscopy (STEM) tomography [15], ellipsometry [16], grazing incidence small angle X-ray scattering (GISAXS) [8], and grazing incidence small angle neutron scattering (GISANS) [17]. Furthermore, specular neutron reflectometry has previously been reported to characterize layer thickness, roughness and chemical composition before and after SIS of MH and PS-OH [10]. By reflecting a neutron beam at a sample, and detecting the reflected intensity in various angles, the data can be fitted to a model of the sublayers [18].

Previously reported results showed the effect of number of infiltration cycles in dynamic infiltration using multiple pulses of TMA/H_2_O per cycle into MH and PS-OH, respectively, as well as the infiltration selectivity, and sub-10 nm pattern transfer into silicon using an infiltrated PS-*b*-MH template [10]. The maximum infiltrated amount of infiltrated Al_2_O_3_ into MH was then found to be 23 vol%, which was sufficient to perform a pattern transfer to an aspect ratio of 2:1. Initial investigation of semi-static infiltration using single pulses per cycle was also reported, indicating the possibility of a more efficient use of precursor material. However, apart from the number of infiltration cycles, the previous study provided no information on the influence of process parameters on the degree of infiltration. A higher degree of infiltration could be important to enable an increase in the possible aspect ratio in pattern transfer. In this study, the effect of the semi-static SIS process parameters temperature, precursor exposure time, and precursor pulse duration for TMA/H_2_O infiltration into MH and PS-OH using specular neutron reflectometry is studied, aiming for further understanding of the reaction mechanisms, as well as to serve as basis for optimization of the process of following sub-10 nm pattern transfer of carbohydrate-based BCPs. As proof of concept, semi-static infiltration of TMA/H_2_O into PS-*b*-MH is thereafter used to create a 12 nm pitch etch mask for pattern transfer into silicon.

## 2. Results and Discussion

**Figure 1:**
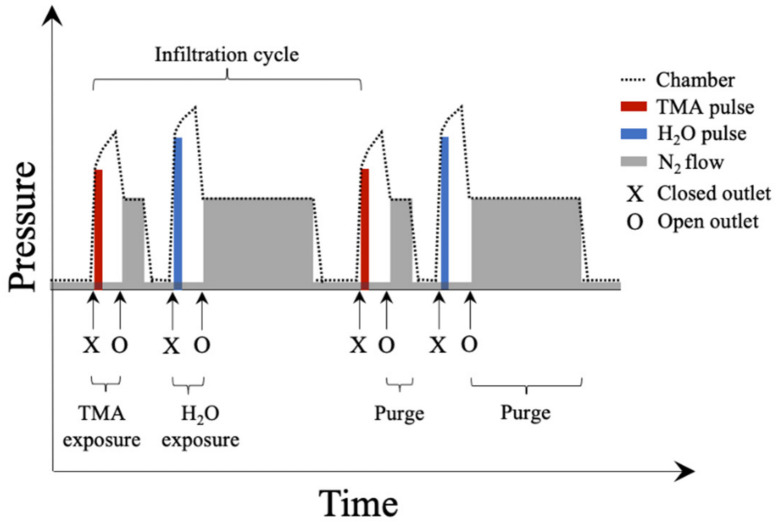
Schematic illustration of chamber pressure as a function of time in semi-static sequential infiltration synthesis (SIS). Not to scale. Adapted with permission from [10], copyright 2021 the Authors. Published by ACS Publications.

In the semi-static SIS employed, the chamber outlet in an atomic layer deposition (ALD) tool was closed just before a TMA precursor pulse was introduced, and a low flow of nitrogen gas was maintained into the chamber during the precursor exposure to avoid back flow contamination (see Figure 1). After the duration of the TMA exposure had passed, the chamber outlet was opened, and the nitrogen flow was increased for the purge of excess precursors and byproducts. The flow was thereafter lowered, to prepare for the second half of the infiltration cycle. The chamber outlet was closed, just before introducing the water (H_2_O) precursor. After the water exposure time duration had passed, the chamber outlet was opened, and the nitrogen flow increased. The excess precursor and byproducts were thereby purged. The flow was once again lowered, and the system ready for any subsequent infiltration cycles. This study used two semi-static infiltration cycles for the investigation of process parameters.

To evaluate the infiltration selectivity between the homopolymers MH and PS-OH for different SIS process parameters, thin films of each type were spin-coated separately onto 1 mm thick silicon substrates. The samples were studied using specular neutron reflectometry (NR) in air at a monochromatic wavelength *λ* of 5.21 Å at the Swedish collaborative research group (CRG) instrument Super-ADAM, Institute Laue-Langevin (ILL), Grenoble, France [19,20,21]. The neutrons interact with the nuclei of the sample and scatter. The reflectivity *R*, which is the intensity ratio of reflected neutron beam to incident beam, is in specular NR measured where the angle of reflection is equal to the incident angle, and *R* was here measured as a function of grazing angle *θ* spanning from 0 to 4°. As *θ* is related to the neutron momentum transfer component perpendicular to the sample surface *Q*_z_ according to

(1) $$Q_{z}=\frac{4\pi }{\lambda }\cdot \sin\theta ,\;$$

*R* is usually plotted as a function of *Q*_z_ [22]. The data [19] was reduced by subtracting background, normalizing the reflected intensity to direct beam, and correcting for overillumination using *pySAred* software [23]. Simulations of specular reflectivity using an optical slab model in *GenX* software were then fitted to the data [24]. The model included, e.g., thickness, top roughness, and scattering length density (SLD) for each sublayer. The SLD is related to material density *ρ*, molar mass *M*, and neutron bound coherent scattering length *b*_c_ [25], and can be calculated from

(2) $$\mathrm{SLD}=N_{A}\cdot \rho \cdot \frac{\sum_{i}b_{c,i}}{\sum_{i}M_{i}},\;$$

summarized for all constituent elements *i*, where *N*_A_ is the Avogadro constant (see Table 1). It can be noted that air is considered to have a neutron SLD of zero. The neutron bound scattering length *b*_c_ is an isotope-specific parameter, hence the chemical composition of the material also can be derived from the analysis [26,27]. A bare silicon substrate was modelled to have an infinite thickness and a SLD of 2.07⋅10^−6^ Å^−2^, and was measured to have a roughness of 3 Å, and a native oxide layer atop of 2 Å thickness, 2 Å roughness, and a SLD of 4.16⋅10^−6^ Å^−2^ (see Supplementary Materials, Figure S1). These were thereafter used as constant values in the NR modelling of thin films on top of substrates. The SLD of a mixture of two components, A and B, can, from Equation (2), be expressed as

SLD_mix_ = *x*⋅SLD_A_ + (1 − *x*)⋅SLD_B_, (3)

which then enables calculation of the volume fraction *x* of component A.

In SIS of block copolymers, the temperature is often chosen to be below the glass transition temperature *T*_g_ of respective block. Sun et al. found no obvious glass transition temperature for MH [28], although caramelization occurs at 190 °C [29]. Poly(styrene) has its *T*_g_ at about 100 °C [30], hence the maximum temperature was chosen to be 100 °C.

### 2.1. Process Parameter Effect on MH Infiltration

The MH was spin-coated onto a silicon substrate, and the pristine layer was measured and analyzed using specular NR to have a thickness of 120 Å, a roughness of 5 Å, and an SLD of 1.56⋅10^−6^ Å^−2^ (see Table 2). This SLD value was thereafter used to represent pristine MH in subsequent modelling, and it was concluded that a model could be fitted to the data if the infiltrated polymer layer was divided into two sublayers.

**Figure 2:**
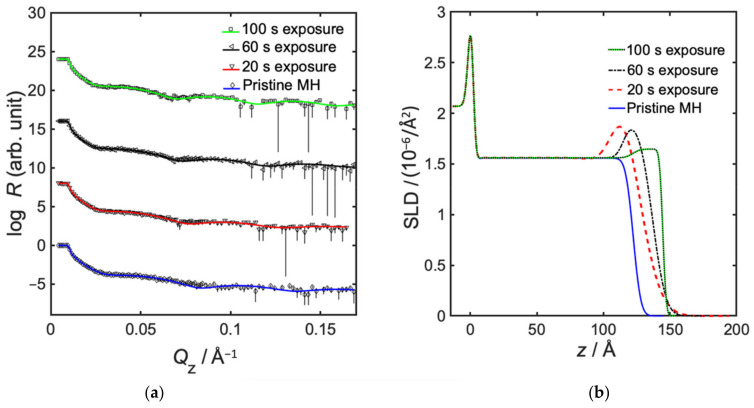
Neutron reflectometry data, showing effect of exposure time in semi-static infiltration of TMA/H_2_O into MH. (**a**) Neutron specular reflectivity profiles, showing measured data with error bars, and fitted models as solid lines; and (**b**) fitted neutron SLD as a function of distance from substrate, where z = 0 represents the interface between silicon and native oxide.

Semi-static infiltration of TMA/H_2_O into MH for exposure times ranging from 20 to 100 s was investigated. The alumina content in MH was here shown to decrease with increasing exposure time, where 20 s resulted in a hybrid material corresponding to an inclusion of 40 vol% pure Al_2_O_3_ (see Figure 2 and Table 2). The decreasing alumina content with increasing exposure time may be caused by the fact that the precursors in the chamber are being more diluted with nitrogen with time, therefore less free precursors exist in the sample at long exposure times. It could also be due to increased desorption at longer exposure times. Furthermore, the infiltration depth was decreasing with decreasing exposure time, to be only 14 Å for 20 s exposure. Another observation is that the total thickness of both non-infiltrated lower layer and infiltrated top layer of the infiltrated samples was decreasing with decreasing exposure time, from 143 to 121 Å (see Table 2).

**Figure 3:**
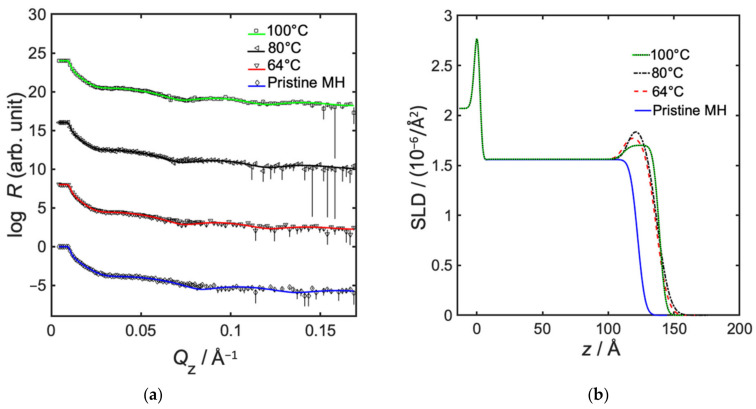
Neutron reflectometry data, showing effect of temperature in semi-static infiltration of TMA/H_2_O into MH. (**a**) Neutron specular reflectivity profiles, showing measured data with error bars, and fitted models as solid lines, and (**b**) fitted neutron SLD as a function of distance from substrate, where z = 0 represents the interface between silicon and native oxide.

The amount of incorporated Al_2_O_3_ in MH from infiltration was also studied as a function of temperature, and a maximum was indicated somewhere between 64 and 100 °C, corresponding to 8 vol% inclusion of Al_2_O_3_ and 22 Å infiltration depth at 80 °C (see Figure 3 and Table 3). It should, however, be noted that the difference in SLD between 64 and 80 °C lies within the margin of error, and that the difference between 80 and 100 °C was just outside the error margin. The temperature influences the diffusion of precursors within the polymer, where a particle normally diffuses more easily at a higher temperature [31]. Additionally, the infiltration efficiency is related to the energy levels of the possible molecular reactions [11]. The result of a small maxima is in line with the findings of Weisbord et al., who concluded that there is a relation between maximum mass gain during infiltration at thermal equilibrium, and having equal forward and reverse reaction rates for the Lewis pair coordination between precursor and polymer functional group [9]. At this point there is equilibrium, and Gibbs free energy change Δ*G* is zero. When Gibbs free energy is negative, the forward reaction rate is higher, and when it is positive the reverse reaction dominates [9]. Furthermore, Weisbord et al. stated that, at a lower process temperature and a negative Δ*G*, the high forward reaction rate further hindered diffusion, and the infiltration depth was lower, whereas at a higher process temperature, and a positive Δ*G*, the high reverse reaction rate further promoted deep infiltration, at the expense of lost coordination bonds [9]. In this study, however, the differences in infiltration depth with temperature were within the error margins, therefore no conclusions could be drawn.

**Figure 4:**
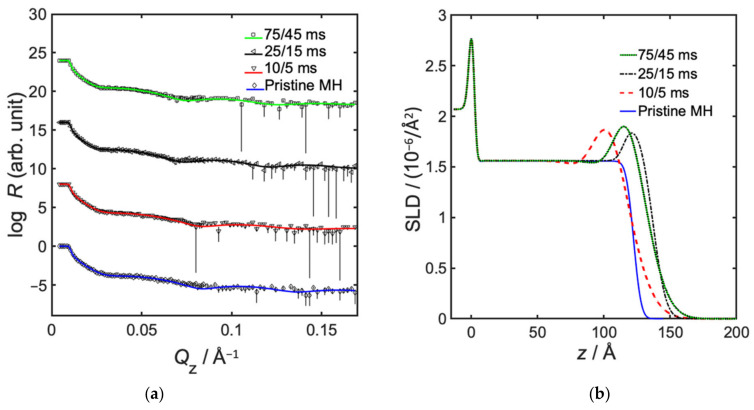
Neutron reflectometry data, showing effect of precursor pulse duration in semi-static infiltration of TMA/H_2_O into MH. (**a**) Neutron specular reflectivity profiles, showing measured data with error bars, and fitted models as solid lines, and (**b**) fitted neutron SLD as a function of distance from substrate, where z = 0 represents the interface between silicon and native oxide.

The precursor pulse duration is directly related to the precursor partial pressure in the infiltration chamber. Results show that a minimum degree of infiltration occurs somewhere between 10 and 75 ms TMA pulse. Equally high degrees of infiltration, corresponding to 40 vol% Al_2_O_3_ incorporation, was measured at the shorter and at the longer precursor pulses, but again at the expense of a decreased infiltration depth, which was only about 15 Å (see Figure 4 and Table 4). A longer pulse duration of 75 ms TMA and 45 s H_2_O leads to a higher precursor partial pressure as more material is introduced into the environment. This should promote a higher degree of infiltration. However, a high number of covalently bonded molecules might then hamper diffusion, and the infiltration depth may therefore be reduced [31]. Regarding the shorter precursor pulses of 10 ms TMA and 5 ms H_2_O, we can only speculate that a more deposition-like regime is entered. Further studies would be required to investigate the involved mechanisms.

### 2.2. Process Parameter Effect on PS-OH Infiltration

**Figure 5:**
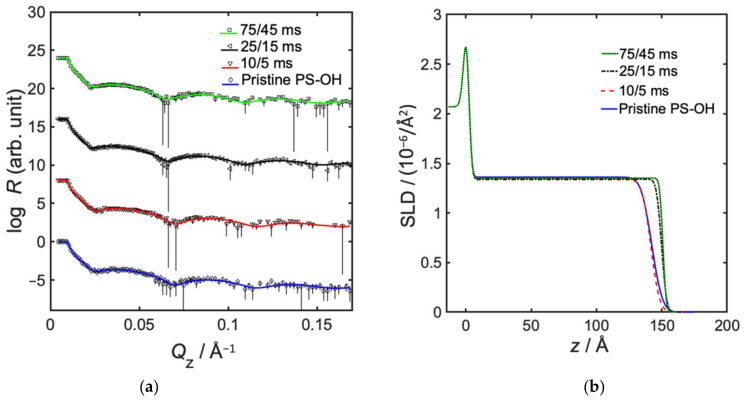
Neutron reflectometry data, showing effect of precursor pulse duration in semi-static infiltration of TMA/H_2_O into hydroxyl-terminated poly(styrene) (PS-OH). (**a**) Neutron specular reflectivity profiles, showing measured data with error bars, and fitted models as solid lines, and (**b**) fitted neutron SLD as a function of distance from substrate, where z = 0 represents the interface between silicon and native oxide.

The PS-OH was spin-coated onto a silicon substrate, then measured and analyzed using specular NR to 140 Å thickness, 6 Å roughness, and a SLD of 1.36⋅10^−6^ Å^−2^ (see Figure 5 and Table 5). When fitting models to the data, the infiltrated PS-OH layer could be modelled as one layer. A high infiltration selectivity between MH and PS-OH have previously been reported for other SIS processes [10]. The effect of process parameters on PS-OH was investigated to ensure preservation of infiltration selectivity. The precursor exposure time had no effect on the corresponding Al_2_O_3_ content of PS-OH, as exposure times ranging from 20 to 100 s resulted in no measurable infiltration (see Supplementary Materials, Figure S2 and Table S1). The change of temperature showed no effect on PS-OH infiltration between 64 and 100 °C, as none could be measured (see Supplementary Materials, Figure S3 and Table S2). Neither at the different precursor pulses of TMA and H_2_O into PS-OH, any inclusion of Al_2_O_3_ was measured (see Figure 5 and Table 5). The here analyzed PS-OH includes the hydroxyl group, and it should be noted that the infiltration selectivity to PS should be higher, as it is lacking functional groups to react with the TMA, unless contamination or trapping of precursors occurs [16].

### 2.3. Infiltration into PS-b-MH for Pattern Transfer

**Figure 6:**
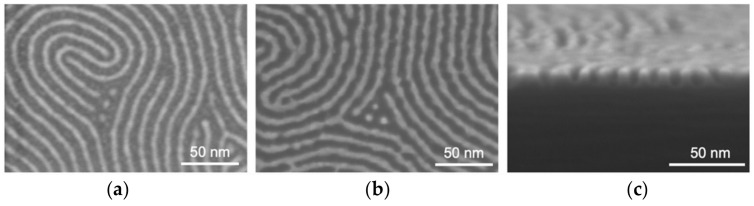
Pattern transfer of 12 nm pitch features. (**a**) scanning electron microscopy (SEM) top view image of a PS-*b*-MH layer on silicon after semi-static TMA/H_2_O infiltration and polymer removal, (**b**) SEM top view image of features etched into silicon, and (**c**) SEM cross-section image of features etched into silicon.

To verify the concept of using a semi-static infiltration method to fabricate etch masks for pattern transfer, a thin film of PS-*b*-MH was self-assembled on top of a silicon substrate using a method reported elsewhere [10]. The film was semi-statically infiltrated with four cycles at 80 °C, using 75/45 ms TMA/H_2_O pulses, and 20 s precursor exposure, resulting in a hybrid material with alumina in the MH block. The PS matrix was thereafter removed by reactive ion etching (RIE) in oxygen plasma and the remaining hybrid features were inspected using scanning electron microscopy (SEM) (see Figure 6a). These alumina-containing hybrid features then acted as an etch mask in RIE in a fluoro-based plasma, to transfer the sub-10 nm pattern at 12 nm pitch into the underlying silicon substrate to an aspect ratio of approximately 2:1, as can be seen in Figure 6b,c. Having established the concept and concluded that the etching performance should be minimum comparable to the dynamic infiltration method described in [10], motivates further investigation and optimization of semi-static infiltration into MH-based block copolymer for pattern transfer.

## 3. Methods/Experimental

The methods/experimental section has been divided into sample preparation, sequential infiltration synthesis, neutron reflectometry characterization, and pattern transfer.

### 3.1. Sample Preparation

Substrates were diced to 35 × 35 mm^2^ Si(111) of 1 mm thickness. 1 wt% maltoheptaose (MH, 1.2 kg/mol, Hayashibara Co., Ltd., Okayama, Japan) was dissolved in deionized water and isopropanol (3:1, *v*/*v*). The substrates were cleaned in acetone and isopropanol, baked on a hotplate at 200 °C for minimum 4 min, thereafter, treated in ozone (UVOH-150, FHR Anlagenbau GmbH, Ottendorf-Okrilla, Germany) for 3 min. The substrates were immediately spin-coated at 5000 rpm, and baked on a hotplate at 80 °C for 3 min. Hydroxyl-terminated poly(styrene) (PS−OH, 4.5 kg/mol, PDI 1.06) was anionically polymerized as reported elsewhere [10], and then dissolved in anisole to 1 wt%. The substrates were thereafter pre-treated, spin-coated with the PS-OH mixture, and baked in the same manner as previously described for MH.

The spin-coated MH layer was measured and analyzed using a Cauchy model in variable angle spectroscopic ellipsometry (VASE) (RC2, J.A. Wollam, Co., Inc., Lincoln, NE, USA) to 122 Å thickness. The spin-coated PS-OH layer was also measured with VASE, analyzed using a Cauchy model, to 138 Å thickness.

### 3.2. Sequential Infiltration Synthesis

The semi-static SIS was performed in a Savannah S100 ALD equipment (Veeco, Plainview, USA) at 5 standard cubic centimeters per minute (sccm) N_2_ flow. For a standard procedure, two cycles of the following sequence were performed. The outlet valve was closed, and the precursor trimethyl aluminum (TMA) released for 25 ms. After an exposure time of 60 s, the outlet valve was opened. The flow was thereafter increased to 100 sccm for 60 s purging of excess material and byproducts. The outlet was closed, and after 120 s at 5 sccm N_2_, the precursor H_2_O was released for 15 ms. After 60 s exposure, the outlet valve was opened, and the chamber purged at 100 sccm for 180 s. The last step of the cycle was 120 s at 5 sccm N_2_. However, for process parameter investigation the following parameters were used, maintaining other parameters as standard: the temperatures 64 °C, and 100 °C, respectively; the precursor TMA/H_2_O pulse durations 10 ms/5 ms, and 75 ms/45 ms, respectively; and the TMA/H_2_O exposure times 20 s/20 s, and 100 s/100 s, respectively.

### 3.3. Neutron Reflectometry Characterization

At Super-ADAM, Institut Laue–Langevin (ILL), Grenoble, France, a monochromatic beam at 5.21 Å was used to perform specular neutron reflectometry in air. By scanning impinging neutron beam at grazing angle from 0 to 4.0° the neutron momentum transfer perpendicular to the sample surface *Q*_z_ ranged from 0 to 0.17 Å^−1^. Raw data were reduced in *pySAred* software by subtracting background, normalizing the reflected intensity to that of the direct beam, and correcting for overillumination [23]. The software *GenX* was used to fit an optical slab model to the NR data, to provide information on thickness, roughness, and chemical composition of each sublayer [24]. The derived neutron scattering length density (SLD) is a function of the material density, the neutron bound coherent scattering length (unique for each type of isotope), and molar mass. Therefore, it was possible to calculate the volumetric percentage of included Al_2_O_3_ in the polymer, by comparing the SLD before and after infiltration.

### 3.4. Pattern Transfer

Silicon substrates, 15 × 15 mm^2^ of 0.525 mm thickness, were spin-coated to a thickness of 11 nm using a solution of 0.75 wt% poly(styrene)-*block*-maltoheptaose (4.5k-*b*-1.2k) in anisole. The polymer was synthesized, and the film self-assembled in vapor of tetrahydrofuran and water, as described in [10]. Semi-static SIS was performed as previously, but for 4 cycles in 80 °C, with 75 ms/45 ms TMA/H_2_O pulse durations, and 20 s exposure time. The poly(styrene) matrix was thereafter removed in inductively coupled plasma reactive ion etching (ICP-RIE) (Apex SLR, Plasma-Therm, Saint Petersburg, USA) in 20 °C, 3 mTorr, 30 sccm O_2_, 25 W RF power, 10 W ICP power for 135 s. The silicon etching was performed in the same tool using 26/54/20 sccm SF_6_/C_4_F_8_/Ar, 5 mTorr, 25 W RF, 300 W ICP, at 20 °C for 45 s. The inspection was performed in SEM (SU8010 Hitachi, Ltd., Tokyo, Japan).

## 4. Conclusions

Neutron reflectometry is an excellent tool to evaluate the degree of infiltration from SIS to create hybrid materials. It is especially powerful for nanotechnology, as the technique fills a gap which arises when traditional characterization techniques, such as SEM and energy-dispersive X-ray spectroscopy, seem too coarse. The process parameters exposure time, and precursor pulse duration are highly relevant in semi-static SIS of TMA/H_2_O into MH, and there is a trade-off to be made between infiltration depth and alumina content. The amount of included Al_2_O_3_ into MH could here be increased to up to 40 vol%, which is a significant increase from the previously reported maximum of 23 vol% [10], especially considering that the amount of TMA precursor material was reduced up to approximately 600 times. The infiltration selectivity to PS-OH was preserved for all investigated process parameters. To ensure a high degree of infiltration into MH, longer pulse duration of 75 ms TMA and 45 ms H_2_O, and shorter exposure times of 20 s of each precursor at about 80 °C in semi-static SIS could be a good choice, although further investigation would likely improve the understanding of the interplay between infiltration parameters and resulting etch performance. The infiltration mechanisms are complex, evident from the fact that maxima or minima could be found in degree of infiltration when varying only one process parameter. By increasing the amount of alumina in the hybrid material, the etch selectivity between mask and underlying material can be increased, which should enable a higher aspect ratio pattern transfer from carbohydrate-based high-*χ* material, such as 12 nm pitch PS-*b*-MH. By establishing that semi-static infiltration of TMA/H_2_O into PS-*b*-MH can be used to fabricate alumina-containing etch masks for 12 nm pitch pattern transfer into silicon, demonstrating an aspect ratio of 2:1, the method can be concluded to be one of relatively few studies showing pattern transfer at this high pattern density.

## Figures and Tables

**Table 1:** Calculated scattering length density SLD from theoretical density *ρ*, neutron bound coherent scattering length *b*_c_ [26,27], and molar mass *M* for relevant materials.

Material	*ρ*/10^3^ (kg/m^3^)	∑*_i_b_c,i_*/10^−15^ m	*M*/10^−3^ (kg/mol)	SLD/10^−6^ Å^−2^
Si	2.33	4.15	28.1	2.07
SiO_2_	2.65	15.8	60.1	4.18
Al_2_O_3_	3.95	24.3	102	5.67
PS (C_8_H_8_)_n_	1.00	23.3	104	1.35
MH (C_42_H_72_O_36_)	1.85	219	1152	2.12

**Table 2:** Effect of exposure time in semi-static infiltration of trimethyl aluminum/water (TMA/H_2_O) into maltoheptaose (MH).

MH	Pristine	Infiltrated		
Exposure Time/s	0	20	60 *	100
Thickness top layer/Å	-	13.8 ± 0.8	22 ± 3	23 ± 3
SLD top layer/10^−6^ Å^−2^	-	3.2 ± 0.2	1.9 ± 0.1	1.6 ± 0.1
Top roughness top layer/Å	-	14 ± 1	8 ± 2	2 ± (+6/−1)
Included Al_2_O_3_/vol%	0	40	8	2
Thickness lower layer/Å	120 ± 3	107 ± 3	113 ± 3	120 ± 3
SLD lower layer/10^−6^ Å^−2^	1.56 ± 0.03	1.56	1.56	1.56
Top roughness lower layer/Å	5 ± 2	9	4	4
Thickness native oxide layer/Å	2	2	2	2
SLD native oxide layer/10^−6^ Å^−2^	4.16	4.16	4.16	4.16
Top roughness native oxide layer/Å	2	2	2	2
SLD silicon substrate layer/10^−6^ Å^−2^	2.07	2.07	2.07	2.07
Top roughness silicon substrate layer/Å	3	3	3	3

* Reference sample.

**Table 3:** Effect of temperature in semi-static infiltration of TMA/H_2_O into MH.

MH	Pristine	Infiltrated		
Temperature/°C	-	64	80 *	100
Thickness top layer/Å	-	25 ± 3	22 ± 3	27 ± 2
SLD top layer/10^−6^ Å^−2^	-	1.79 ± 0.09	1.9 ± 0.1	1.70 ± 0.06
Top roughness top layer/Å	-	7 ± 2	8 ± 2	4 ± 3
Included Al_2_O_3_/vol%	0	6	8	3
Thickness lower layer/Å	120 ± 3	109 ± 3	113 ± 3	110 ± 2
SLD lower layer/10^−6^ Å^−2^	1.56 ± 0.03	1.56	1.56	1.56
Top roughness lower layer/Å	5 ± 2	4	4	4
Thickness native oxide layer/Å	2	2	2	2
SLD native oxide layer/10^−6^ Å^−2^	4.16	4.16	4.16	4.16
Top roughness native oxide layer/Å	2	2	2	2
SLD silicon substrate layer/10^−6^ Å^−2^	2.07	2.07	2.07	2.07
Top roughness silicon substrate layer/Å	3	3	3	3

* Reference sample.

**Table 4:** Effect of precursor pulse duration in semi-static infiltration of TMA/H_2_O into MH.

MH	Pristine	Infiltrated		
Pulse Duration (TMA/H_2_O)/ms	-	10/5	25/15 *	75/45
Thickness top layer/Å	-	16.3 ± 0.7	22 ± 3	15.1 ± 0.9
SLD top layer/10^−6^ Å^−2^	-	3.22 ± 0.2	1.9 ± 0.1	3.21 ± 0.2
Top roughness top layer/Å	-	17.6 ± 0.9	8 ± 2	16 ± 1
Included Al_2_O_3_/vol%	0	40	8	40
Thickness lower layer/Å	120 ± 3	93 ± 3	113 ± 3	109 ± 3
SLD lower layer/10^−6^ Å^−2^	1.56 ± 0.03	1.56	1.56	1.56
Top roughness lower layer/Å	5 ± 2	10	4	9
Thickness native oxide layer/Å	2	2	2	2
SLD native oxide layer/10^−6^ Å^−2^	4.16	4.16	4.16	4.16
Top roughness native oxide layer/Å	2	2	2	2
SLD silicon substrate layer/10^−6^ Å^−2^	2.07	2.07	2.07	2.07
Top roughness silicon substrate layer/Å	3	3	3	3

* Reference sample.

**Table 5:** Effect of precursor pulse duration in semi-static infiltration of TMA/H_2_O into PS-OH.

PS-OH	Pristine	Infiltrated		
Pulse Duration (TMA/H_2_O)/ms	-	10/5	25/15 *	75/45
Thickness top layer/Å	140 ± 2	140 ± 3	148 ± 2	148 ± 2
SLD top layer/10^−6^ Å^−2^	1.36 ± 0.03	1.34 ± 0.04	1.34 ± 0.03	1.35 ± 0.03
Top roughness top layer/Å	6 ± 4	5 ± 5	3 ± 3	2 ± (+4/−2)
Included Al_2_O_3_/vol%	0	0	0	0
Thickness native oxide layer/Å	2	2	2	2
SLD native oxide layer/10^−6^ Å^−2^	4.16	4.16	4.16	4.16
Top roughness native oxide layer/Å	2	2	2	2
SLD silicon substrate layer/10^−6^ Å^−2^	2.07	2.07	2.07	2.07
Top roughness silicon substrate layer/Å	3	3	3	3

* Reference sample.

## Data Availability

Specular neutron reflectivity data can be found in Löfstrand, A.; Vorobiev, A. Investigation of Static Sequential Infiltration Synthesis into Polystyrene-block-Maltoheptaose by neutron reflectivity. Institut Laue-Langevin (ILL): Grenoble, 2021; doi:10.5291/ILL-DATA.CRG-2762.

## Supplementary Materials

The following are available online at https://www.mdpi.com/article/10.3390/polym14040654/s1, Figure S1: Neutron reflectometry data of bare silicon substrate, Figure S2: Neutron reflectometry data, showing effect of precursor exposure time in semi-static infiltration of TMA/H_2_O into PS-OH, Table S1: Effect of precursor exposure time in semi-static infiltration of TMA/H_2_O into PS-OH, Figure S3: Neutron reflectometry data, showing effect of temperature in semi-static infiltration of TMA/H_2_O into PS-OH, Table S2: Effect of temperature in semi-static infiltration of TMA/H_2_O into PS-OH, Figure S4: SEM images of hybrid features from semi-static infiltration of TMA/H_2_O into PS-*b*-MH, and Figure S5: SEM raw data images of hybrid feature etch mask and features etched into silicon (seen in Figure 6).
