# Supplementary material for: Sequential Infiltration Synthesis into Maltoheptaose and Poly(styrene): Implications for Sub-10 nm Pattern Transfer
Source: Polymers (Basel). 2022 Feb 10;14(4):654. doi: 10.3390/polym14040654 (PMC8878060; doi:10.3390/polym14040654)
Supplement: Supplementary file 1 [file polymers-14-00654-s001.zip › polymers-1515432-supplementary.pdf]

Supporting Information

Sequential Infiltration Synthesis into Maltoheptaose and Poly(styrene): Implications for Sub-10 nm Pattern Transfer

Löfstrand, A.[1], Vorobiev, A.[2], Mumtaz, M.[3], Borsali, R.[3], and Maximov. I[1].

[1] NanoLund and Solid State Physics, Lund University, SE-221 00 Lund, Sweden;
[2] Division for Materials Physics, Department of Physics and Astronomy, Uppsala University, SE-751 20 Uppsala, Sweden;
[3] Univ. Grenoble Alpes, CNRS, CERMAV, 38000 Grenoble, France.

This supporting information includes specular neutron reflectometry (NR) and analysis of a bare silicon substrate, as can be seen in Figure S1, and in Table S1, and of PS-OH when varying semi-static infiltration precursor exposure time, and temperature, respectively. It also includes scanning electron microscopy (SEM) images of PS-*b*-MH after TMA/H_2O infiltration and PS removal, as well as images of 12 nm pitch features after pattern transfer into silicon using the resulting alumina-containing hybrid etch mask.

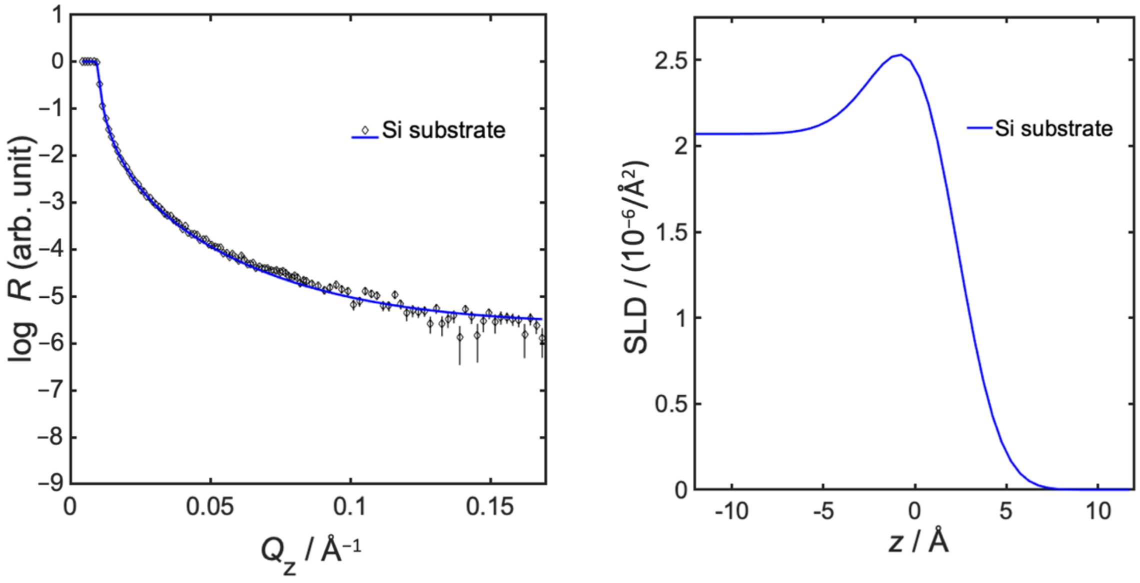

(a) (b)

Figure S1. *Neutron reflectometry data of bare silicon substrate. a) Neutron specular reflectivity profile, showing measured data with error bars, and fitted model as solid line, and b) fitted neutron SLD as a function of distance from substrate, where z=0 represents the interface between silicon and native oxide.*

The effect of precursor exposure time on two cycles of semi-static sequential infiltration synthesis (SIS) of hydroxyl-terminated poly(styrene) (PS-OH) is shown in Figure S2 and Table S1.

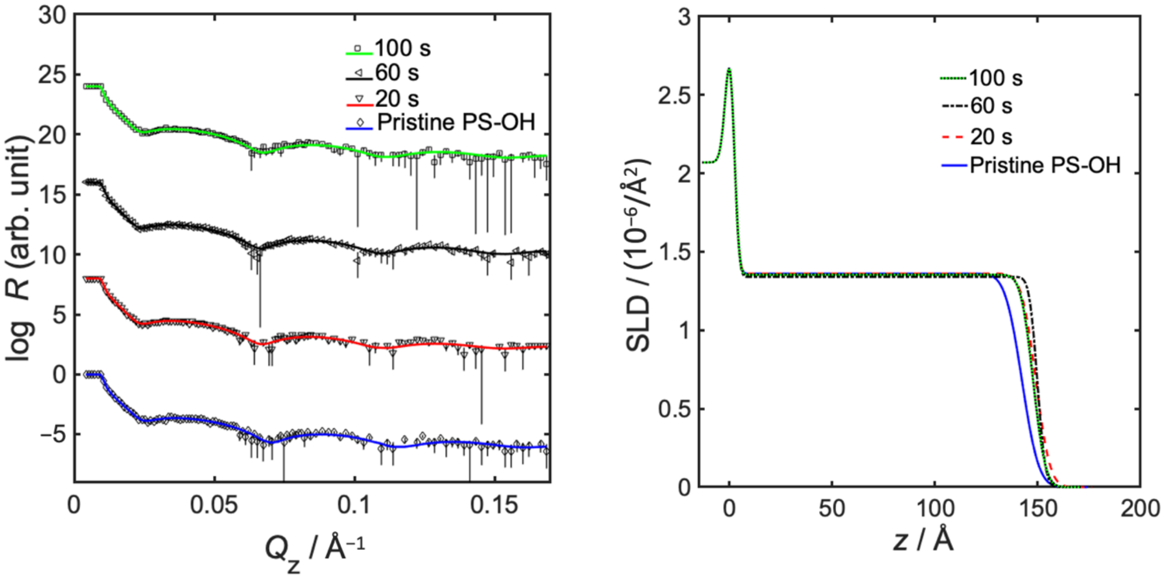

***Figure S2**. Neutron reflectometry data, showing effect of precursor exposure time in semi-static infiltration of TMA/H_2O into PS-OH. a) Neutron specular reflectivity profiles, showing measured data with error bars, and fitted models as solid lines, and b) fitted neutron SLD as a function of distance from substrate, where z=0 represents the interface between silicon and native oxide.*

***Table S1**. Effect of precursor exposure time in semi-static infiltration of TMA/H_2O into PS-OH.*

PS-OH	Pristine		Infiltrated	
Exposure time / s	0	20	60*	100
Thickness top layer / Å	140±2	147±2	148±2	146±2
SLD top layer/ 10^{-6} Å^{-2}	1.36±0.03	1.36±0.03	1.34±0.03	1.36±0.03
Top roughness top layer / Å	6±4	5±4	3±3	5±3
Included Al_2O_3 / vol%	0	0	0	0
Thickness native oxide layer / Å	2	2	2	2
SLD native oxide layer / 10^{-6} Å^{-2}	4.16	4.16	4.16	4.16
Top roughness native oxide layer / Å	2	2	2	2
SLD silicon substrate layer / 10^{-6} Å^{-2}	2.07	2.07	2.07	2.07
Top roughness silicon substrate layer / Å	3	3	3	3

* *Reference sample.*

The effect of temperature on two cycles of semi-static SIS of PS-OH is shown in Figure S3 and Table S2.

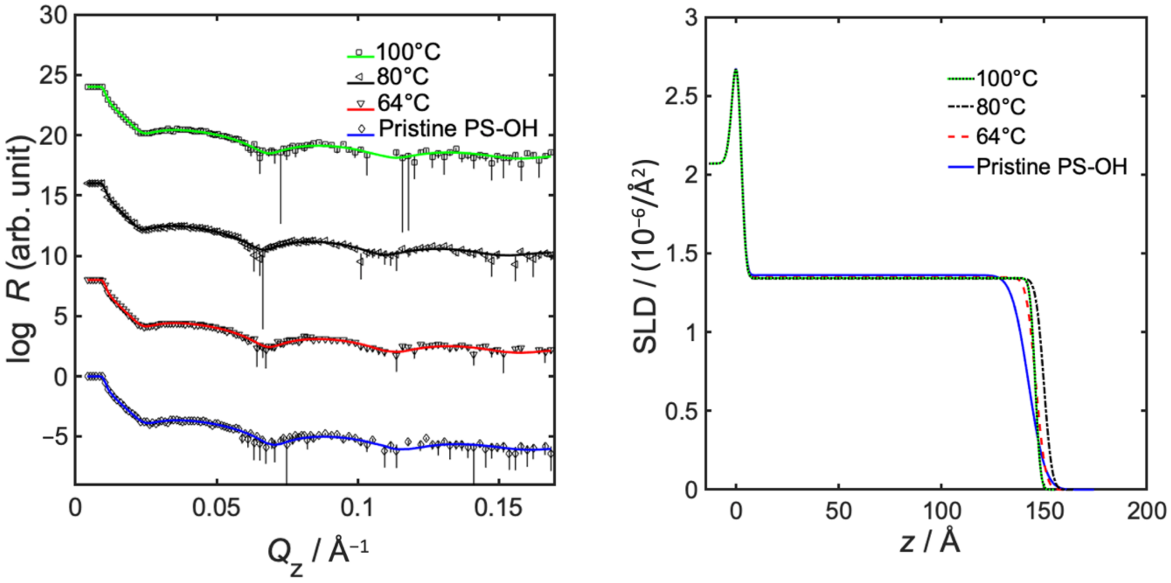

(a) (b)

Figure S3. *Neutron reflectometry data, showing effect of temperature in semi-static infiltration of TMA/H_2O into PS-OH. a) Neutron specular reflectivity profiles, showing measured data with error bars, and fitted models as solid lines, and b) fitted neutron SLD as a function of distance from substrate, where z=0 represents the interface between silicon and native oxide.*

Table S2. *Effect of temperature in semi-static infiltration of TMA/H_2O into PS-OH.*

PS-OH	Pristine		Infiltrated	
Temperature / °C	-	64	80*	100
Thickness top layer / Å	140±2	144±1	148±2	143±2
SLD top layer/ 10^{-6} Å^{-2}	1.36±0.03	1.35±0.02	1.34±0.03	1.34±0.03
Top roughness top layer / Å	6±4	4±3	3±3	2±(+4/-0)
Included Al_2O_3 / vol%	0	0	0	0
Thickness native oxide layer / Å	2	2	2	2
SLD native oxide layer / 10^{-6} Å^{-2}	4.16	4.16	4.16	4.16
Top roughness native oxide layer / Å	2	2	2	2
SLD silicon substrate layer / 10^{-6} Å^{-2}	2.07	2.07	2.07	2.07
Top roughness silicon substrate layer / Å	3	3	3	3

* *Reference sample.*

Semi-static infiltration of TMA/H_2O into an 11 nm layer of the block copolymer poly(styrene)-*block*-maltoheptaose (4.5k-*b*-1.2k) on top of a silicon substrate was investigated. First using the following deviations from the reference process: 20 s exposure time for 2, 4, and 8 cycles, respectively (see Figure S4a, b, and c). Then using the deviations 20 s exposure time, but also 75 ms/45 ms TMA/H_2O pulses, for 2, 4, and 8 cycles, respectively (see Figure S4d, e, and f). SEM inspection was made after poly(styrene) removal in oxygen plasma reactive ion etching.

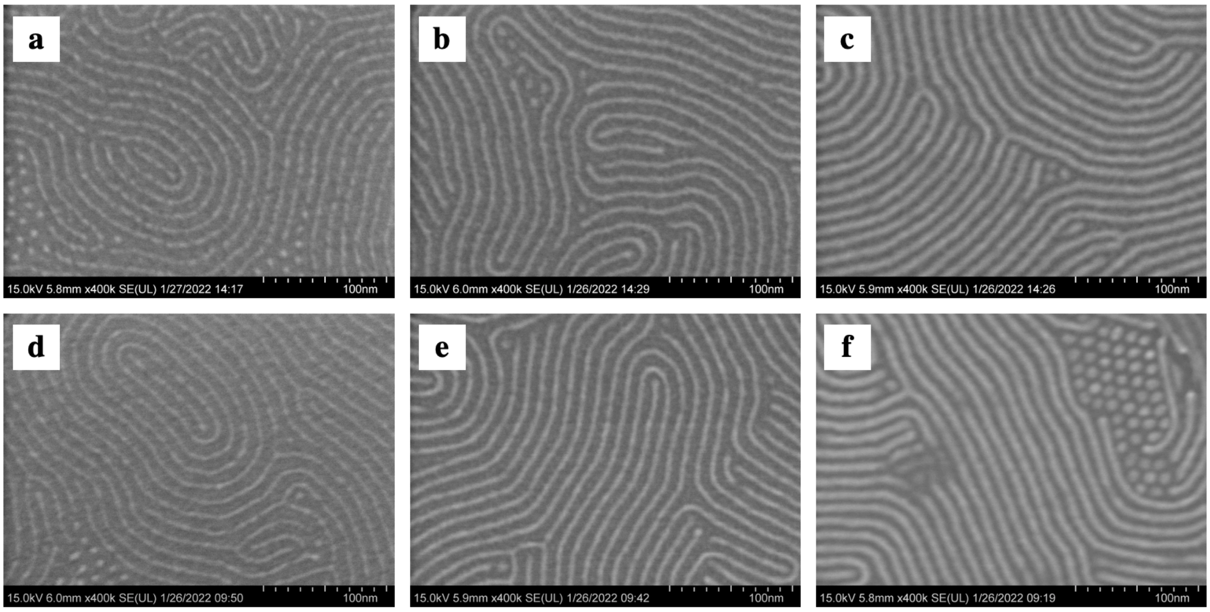

Figure S4. *SEM top view images of semi-statically infiltrated PS-b-MH after poly(styrene) removal. Upper row using 20 s exposure and 25 ms/15 ms TMA/*H_2O *pulses: a) 2 cycles, b) 4 cycles, and c) 8 cycles. Lower row using 20 s exposure and 75 ms/45 ms TMA/*H_2O *pulses: d) 2 cycles, e) 4 cycles, and f) 8 cycles. Some images were adjusted in brightness and contrast to facilitate comparison.*

Figure S5 displays the raw data of the images of etch mask and etched features shown in Figure 6 in the article.

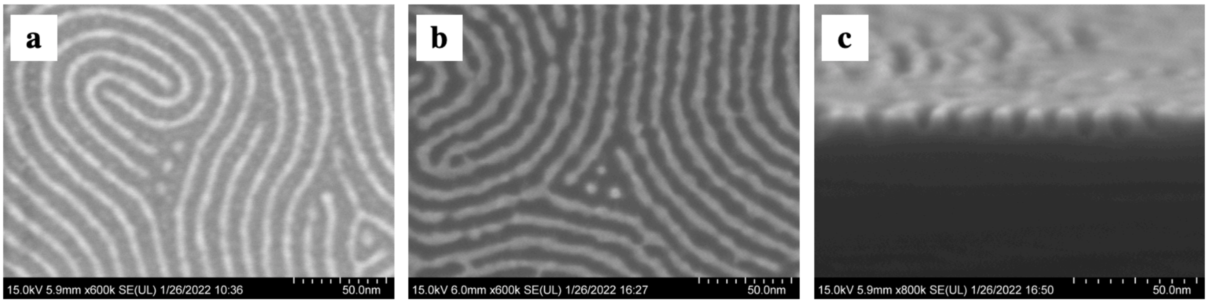

Figure S5. *SEM images, a) top view of semi-statically infiltrated PS-b-MH using 4 cycles of 20 s exposure and 75 ms/45 ms TMA/*H_2O *pulses, as well as poly(styrene) removal, b) top view of features etched into silicon using fluoro-based plasma, and c) cross-sectional view of the same.*
